# A Retrospective Observational Study of the Relationship between Single Nucleotide Polymorphisms Associated with the Risk of Developing Colorectal Cancer and Survival

**DOI:** 10.1371/journal.pone.0117816

**Published:** 2015-02-24

**Authors:** Eva J. A. Morris, Steve Penegar, Nicola Whiffin, Peter Broderick, D. Timothy Bishop, Emma Northwood, Philip Quirke, Paul Finan, Richard S. Houlston

**Affiliations:** 1 Section of Epidemiology and Biostatistics, Leeds Institute of Cancer and Pathology, University of Leeds, Leeds LS9 7TF, United Kingdom; 2 Division of Genetics and Epidemiology, Institute of Cancer Research, Sutton, Surrey, SM2 5NG, United Kingdom; 3 Pathology and Tumour Biology, Leeds Institute of Cancer and Pathology, Level 4 Wellcome Trust Brenner Building, St James’s University Hospital, Leeds LS9 7TF, United Kingdom; 4 John Goligher Colorectal Unit, Leeds Teaching Hospitals, St James’s University Hospital, Beckett Street, Leeds, LS9 7TF, United Kingdom; 5 National Cancer Intelligence Network, 18^th^ Floor Portland House, Bressenden Place, London, SW1E 5RS, United Kingdom; Beijing University of Chemical Technology, CHINA

## Abstract

**Background:**

There is variability in clinical outcome for patients with apparently the same stage colorectal cancer (CRC). Single nucleotide polymorphisms (SNPs) mapping to chromosomes 1q41, 3q26.2, 6p21, 8q23.3, 8q24.21, 10p14, 11q13, 11q23.1, 12q13.13, 14q22, 14q22.2, 15q13.3, 16q22.1, 18q21.1, 19q13.11, 20p12, 20p12.3, 20q13.33 and Xp22 have robustly been shown to be associated with the risk of developing CRC. Since germline variation can also influence patient outcome the relationship between these SNPs and patient survivorship from CRC was examined.

**Methods:**

All enrolled into the National Study of Colorectal Cancer Genetics (NSCCG) were genotyped for 1q41, 3q26.2, 6p21, 8q23.3, 8q24.21, 10p14, 11q13, 11q23.1, 12q13.13, 14q22, 14q22.2, 15q13.3, 16q22.1, 18q21.1, 19q13.11, 20p12, 20p12.3, 20q13.33 and xp22 SNPs. Linking this information to the National Cancer Data Repository allowed patient genotype to be related to survival.

**Results:**

The linked dataset consisted of 4,327 individuals. 14q22.22 genotype defined by the SNP rs4444235 showed a significant association with overall survival. Specifically, the C allele was associated with poorer observed survival (per allele hazard ratio 1.13, 95% confidence interval 1.05–1.22, *P* = 0.0015).

**Conclusion:**

The CRC susceptibility SNP rs4444235 also appears to exert an influence in modulating patient survival and warrants further evaluation as a potential prognostic marker.

## INTRODUCTION

Colorectal cancer (CRC) is a common disease in the UK affecting around 40,000 individuals annually and accounting for 16,000 cancer related deaths each year [1]. Despite major advances in the medical management of CRC over the last 25 years, five-year survival remains at only around 55% [1].

A principle metric of patient prognosis of CRC is stage at presentation [2] however there is significant variability in overall survival (OS) of patients with apparently same stage disease and understanding these differences is clinically important.

There is evidence of familial concordance for survival in a number of cancers, including CRC [3], which suggests that inherited genetic variation can contribute to CRC prognosis. Additionally, studies have reported associations with survival from CRC with genetic variants alone or in combination with specific types of chemotherapy [4–6]. Hence, as a potential prognostic factor the concept of germline variation imparting inter-individual variability in tumour development, progression and metastasis is receiving increased attention [7–11].

Genome-wide association studies (GWAS) have been successful in identifying single nucleotide polymorphisms (SNPs) that are significantly associated with an individual’s risk of developing a CRC [12,13]. In European populations GWAS located CRC susceptibility SNPs have been identified at 1q41, 3q26.2, 5p15.33, 6p21, 8q23.3, 8q24.21, 10p14, 11q13.4, 11q23.11, 12q13.3, 14q22.2, 15q13.3, 16q22.1, 18q21.1, 19q13, 20p12.3, 20q13.33, and Xp22.2 [12–16]. As well as influencing CRC risk, it is entirely plausible these variants may also impact on patient outcome following the diagnosis of CRC.

This hypothesis has been variously examined by a number of researchers but with contradictory results [17–24]. Disparity may be due to the relatively small and heterogeneous cohorts of individuals analysed which had limited power to detect clinically important relationships between SNP genotype and outcome and, hence, the prognostic significance of these CRC susceptibility variants remains controversial. To address shortcomings in previous studies we have made use of the recent linkage [10] of the large National Study of Colorectal Cancer Genetics (NSCCG) [25] with the data in the National Cancer Data Repository (NCDR) [26]. This linkage has offered an opportunity to relate genotype and outcome across a larger population than has previously been possible. Using these data, this study aimed to investigate whether 19 CRC susceptibility SNPs also exerted an influence of survival from the disease.

## MATERIALS AND METHODS

### Patients and record linkage

**Fig 1:**
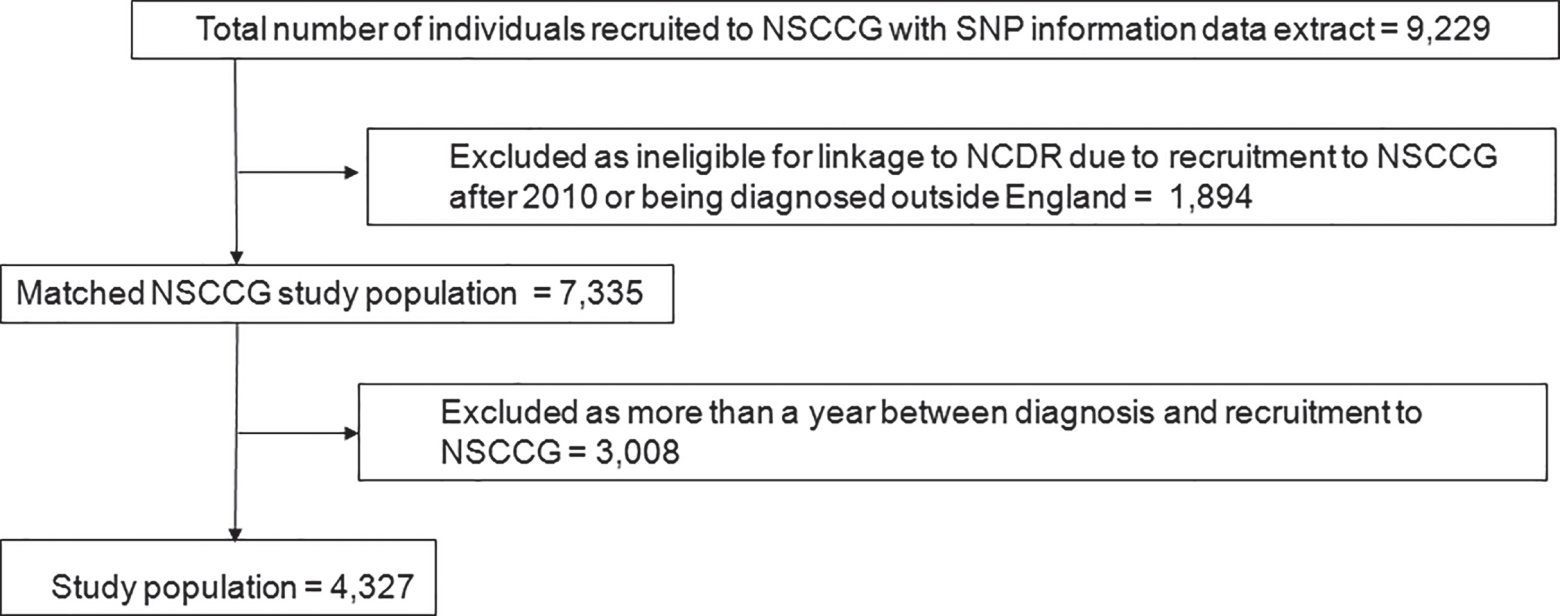
Matching of the NSSCG study cohort and the NCDR.

Full details of the NSCCG have been published elsewhere [25] but, in brief, the study collected DNA and clinicopathological data from over 20,000 individuals with colorectal cancer and a series of spouse/partner controls with the aim of creating a unique resource for the identifying low-penetrance CRC susceptibility genes. All individuals within this study for whom SNP information were available and who could be linked to the NCDR were, therefore, identified and matched using the method described previously [10]. To minimise bias, cases were excluded from the analysis if there was more than a year between the diagnosis of CRC in an individual recorded in the NCDR and their recruitment to the NSCCG (Fig. 1).

All clinical information and biological samples were obtained after fully informed consent was obtained from participating individuals, and in accordance with the tenets of the Declaration of Helsinki. Ethical approval for the both the NSCCG and its linkage to the NCDR were obtained from Multi-Centre Research Ethics Committees (MREC/98/2/67; MREC02/0/97; REC08/S0501/66)

### Genotyping

DNA was extracted from EDTA acid-venous blood samples by conventional methodologies and PicoGreen quantified (Invitrogen Corporation, Carlsbad, California; now Life Technologies). Nineteen SNPs were selected that had been reported to be associated with CRC from 14 chromosomal regions—rs6691170 (1q41), rs10936599 (3q26.2), rs1321311 (6p21), rs16892766 (8q23.3), rs6983267 (8q24.21) rs10795668 (10p14), rs3824999 (11q13), rs3802842 (11q23.1), rs11169552 (12q13.13), rs1957637 (14q22), rs4444235 (14q22.2), rs4779584, (15q13.3), rs9929218 (16q22.1), rs4939827 (18q21.1), rs10411210 (19q13.11), rs4813802 (20p12), rs961253 (20p12.3) rs4925386 (20q13.33) and rs5934682 (xp22). SNP genotyping was performed by allele-specific polymerase chain reaction (LGC Genomics; http://www.kbioscience.co.uk) with primer sequences and conditions available on request. To monitor quality control, we included a set of 136 duplicate samples in assays; genotype concordance was >99.9%. To confirm genotypes, we sequenced 192 samples chosen randomly from cases and controls; concordance between genotypes was 100%.

### Statistical analysis

Statistical analyses were conducted using Stata version 13 (State College, Tx, USA). A *P*-value of 0.05 (two sided) was considered to be significant. When commented, a Bonferroni correction for multiple comparisons corresponded to a value of 0.0026 (0.05/19 SNPs). Results are presented without correction for multiple testing to mitigate against type II error. Differences in patient characteristics between groups were assessed using χ^2^ and Kruskal-Wallis tests. The study end-point was five-year overall survival calculated from date of recruitment to the NSCCG to date of death or when censored (30^th^ June 2011). Kaplan-Meier graphs according to genotype were generated and their homogeneity evaluated using log-rank tests. Cox proportional hazards regression analysis was used to estimate hazard ratios (HR) and their 95% confidence intervals (CI) whilst adjusting for age, sex, Dukes’ stage of disease at diagnosis, deprivation score, tumour site (colon, rectosigmoid junction or rectum), and year of diagnosis. The *P*-values presented correspond to the significance of a test difference among all three of the genotype groups (common allele homozygote, heterozygote and rare allele homozygote).

The power to demonstrate a relationship between SNP genotype and OS was estimated using sample size formulae for comparative binomial trials. To evaluate the chance of obtaining a false-positive association in our data set and to assess the robustness of previously reported associations between SNP genotype and patient outcome, we made use of the false-positive report probability (FPRP) test [27]. The FPRP value is determined by the *P* value, the prior probability for the association, and statistical power. For our analyses, we assumed prior probabilities of 0.05, 0.01 and 0.001; imposing an FPRP cut-off value of 0.5 as advocated [27], values less than 0.5 were considered to be noteworthy, being indicative of a robust association.

Meta-analysis of study findings with previously published data was performed using a fixed-effects model, estimating Cochran’s Q statistic to test for heterogeneity and the *I* ^*2*^ statistic to quantify the proportion of the total variation between studies.

Ethical approval was obtained for both the NSCCG study (MREC/98/2/67; MREC02/0/97) and the linkage and exploitation of the NSCCG and NCDR data (LR/08/S0501/66).

## RESULTS

### Linkage

Information on 9,229 individuals recruited to the NSCCG and with SNP information was supplied for linkage to the NCDR. The study population consisted of 4,327 (46.9%) of these individuals who both matched into the NCDR and who were recruited to the NSCCG within a year of the diagnosis of their disease (Fig. 1).

### Descriptive statistics

Complete clinical and demographic characteristics of the subjects studied are provided in Table 1. The median age at diagnosis of CRC was 60 years (mean, 58.6 years; standard deviation, 8.0). A total of 2,626 cases (60.7%) had colonic, 416 (9.6%) rectosigmoid and 1,285 (29.7%) rectal tumours; the majority of patients presented with Dukes’ stage B and C tumours (3,055, 70.6%).

**Table 1:** The characteristics of the study population.

Characteristic		n	%
Median age at diagnosis (range)		60	(54–65)
Age at diagnosis	≤50	631	14.6
	51–60	1,648	38.1
	>60	2,048	2,048
Sex	Male	2,570	59.4
	Female	1,757	40.6
Self-reported family history of colorectal cancer	No	3,500	80.9
	Yes	827	19.1
Dukes' stage of disease at diagnosis	A	252	5.8
	B	1,171	27.1
	C	1,884	43.5
	D	596	13.8
	Unknown	424	9.8
Tumour site	Colon	2,626	60.7
	Rectosigmoid	416	9.6
	Rectum	1,285	29.7
IMD income category	Most affluent	1,066	24.6
	2	1,016	23.5
	3	979	22.6
	4	739	17.1
	Most deprived	527	12.2
Year of recruitment into NSCCG	2004	789	18.2
	2005	1,489	34.4
	2006	1,073	24.8
	2007	813	18.8
	2008	67	1.6
	2009	50	1.2
	2010	46	1.1
Year of diagnosis of CRC	2003	190	4.4
	2004	1,277	29.5
	2005	1,292	29.9
	2006	1,074	24.8
	2007	366	8.5
	2008	58	1.3
	2009	61	1.4
	2010	9	0.2

Overall, the 5-year survival rate was 64.3% (95%CI 62.9–65.8%). There were 1,658 (38.3%) deaths across the entire cohort. Survival was strongly associated with tumour stage (P<0.0001); 5-year survival ranged from 54.9% (95%CI 0.50–0.60) for patients diagnosed with stage D CRC to 88.4% (95%CI 83.6–91.2%) for those with the stage A CRC. Since these survival rates are not significantly different to those documented in previously published studies investigating the prognosis of actively managed CRC patients [28], it was concluded that there is no evidence that ‘healthy study participant’ selection would bias analyses.

### Relationship between SNP genotype and OS

**Fig 2:**
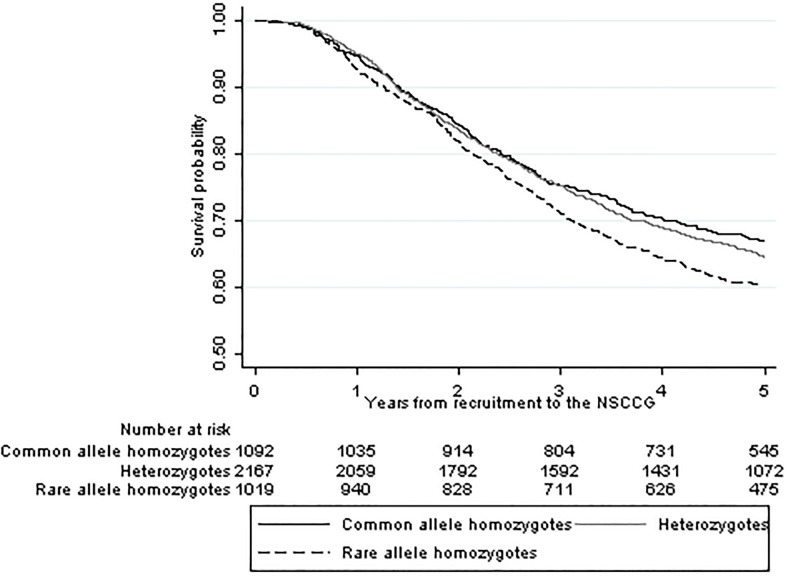
Kaplan Meier survival curves for SNP rs4444235.

There was no statistically significant correlation between SNP genotype and the pathological parameters, site and stage. Only one SNP showed evidence of a correlation with OS (Table 2). A significant association were identified between rs4444235 genotype and prognosis, where the hazards ratio for increasing number of variant alleles was 1.13 (95% CI: 1.05–1.22). Hazard ratios for heterozygosity, homozygosity and carrier status were: 1.18 (95% CI: 1.04–1.34) and 1.28 (95% CI: 1.11–1.48), respectively. It should be noted that the association (*P*_*trend*_ = 0.0015) remained significant if Bonferroni correction for multiple testing was applied (*P*_*adj*_ = 0.032) and remained noteworthy (i.e. FPRP≤0.5) provided the prior was >0.001. Kaplan–Meier estimates (Fig. 2) demonstrated that carriers had lower five-year survival than those with the wild-type genotype (*P*<0.01).

**Table 2:** Unadjusted and risk-adjusted hazard ratios showing the risk of death within five years of diagnosis in relation to SNP status.

SNP	Position (nearest gene)	Risk allele	Number of individuals	Number of deaths	Unadjusted			Adjusted		
					HR	95% CI	P value	HR	95% CI	P value
rs10411210	19q13	C	4,275	1,632	1.02	0.90–1.16	0.76	1.07	0.94–1.22	0.30
rs10795668	10p14	G	4,171	1,600	1.06	0.98–1.15	0.16	1.04	0.96–1.13	0.34
rs10936599	3q26	C	4,285	1,640	1.05	0.96–1.14	0.26	1.07	0.99–1.17	0.10
rs11169552	12q13	C	4,257	1,632	1.01	0.93–1.10	0.75	1.04	0.96–1.14	0.33
rs1321311	6p21	C	4,254	1,626	1.00	0.92–1.08	0.94	0.98	0.90–1.07	0.62
rs16892766	8q23	C	4,270	1,633	0.93	0.82–1.05	0.23	0.94	0.83–1.06	0.32
rs1957637	14q22	A	4,303	1,647	1.00	0.93–1.07	0.98	0.97	0.90–1.04	0.34
rs3802842	11q23	C	4,244	1,625	0.97	0.90–1.05	0.48	0.97	0.90–1.05	0.50
rs3824999	11q13	A	4,263	1,634	0.99	0.92–1.06	0.77	1.02	0.95–1.10	0.58
**rs4444235**	**14q22**	**C**	**4,278**	**1,638**	**1.12**	**1.04–1.50**	**0.001**	**1.13**	**1.05–1.22**	**0.001**
rs4779584	15q13	T	4,251	1,624	1.02	0.94–1.11	0.69	1.01	0.93–1.10	0.85
rs4813802	20p12	G	4,223	1,615	0.98	0.91–1.06	0.59	0.98	0.91–1.06	0.64
rs4925386	20q13	C	4,263	1,630	1.06	0.97–1. 14	0.19	1.09	1.01–1.18	0.03
rs4939827	18q21	T	4,268	1,630	0.97	0.91–1.05	0.47	0.94	0.87–1.01	0.08
rs5934683	Xp22	C	4,225	1,608	0.98	0.92–1.04	0.51	0.96	0.90–1.02	0.14
rs6691170	1q41	T	4,266	1,632	1.01	0.94–1.09	0.75	1.05	0.97–1.13	0.22
rs6983267	8q24	G	4,246	1,623	1.07	0.99–1.12	0.08	1.05	0.98–1.13	0.16
rs961253	20p12	A	4,249	1,624	0.92	0.86–1.00	0.05	0.97	0.90–1.04	0.40
rs9929218	16q22	G	4,271	1,634	1.01	0.94–1.10	0.73	1.01	0.93–1.10	0.76

### Commentary on previously published studies

A number of previous studies have evaluated the relationship of rs4444235 and other risk SNPs with patient prognosis (Table 3). Tenesa *et a l* [21] analysed 10 CRC susceptibility variants but found no association with OS or CRC-specific survival (CSS). Xing *et al* [22] analysed six SNPs in a small cohort of patients in relation to recurrence and death and generated evidence suggesting that rs10795668 (10p14) might influence recurrence (*P* = 0.007, *P*_*adj*_ = 0.042). The effect observed was strongest in those receiving chemotherapy. Phipps *et al* [20] studied 16 CRC SNPs (including some also analysed by Tenesa *et al* [21] and survival in 2,611 CRC patients ascertained from five cohort studies. They reported the 18q21 variant rs4939827 affected OS (*P* = 0.002; *P*_*adj*_ = 0.03). Most recently Abuli and co-workers [17] reported on the relationship between 16 CRC risk SNPs CRC patients requited to the Spanish EPICOLON consortium. Genetic variants rs9929218 at 16q22.1 and rs10795668 at 10p14 were reported to have an effect on OS (*P* = 0.0179 and 0.057, respectively) albeit neither robust after adjustment for multiple testing (*P*_*adj*_ = 0.28 and 0.91, respectively). Most recently, Hoskins *et al* [19] have reported on the relationship between 11 SNPs and survival. The only associations reported to be significant was for homozgosity for 8q24 SNPs rs7013278, rs7014346 (*P* = 0.01 and 0.03 respectively, *P*_*adj*_ for number of risk loci = 0.06 and 0.18 respectively). Contemporaneously Dai and co-workers [18] reported on the relationship between 26 SNPs in 10 of the GWAS risk loci in a cohort restricted to individuals with Dukes’ stage B and C cancers. rs961253 (20p12.3), rs355527 (20p12.3), rs4464148 (18q21.1), rs6983267 (8q24.21) and rs10505477 (8q24.21) were significantly associated with survival. The effects were no longer statistically significant, however, after adjustment for multiple testing. Irrespective of correction for multiple testing assuming a prior of 0.001 none of these associations are inherently robust.

**Table 3:** Findings of other studies investigating the association of CRC susceptibility SNPs with prognosis.

Study	Cohort size	Outcomes assessed	SNPs investigated																																								
			rs10318	rs10411210	rs10505477	rs10749971	rs10795668	rs10808555	rs10936599	rs11169552	rs11213809	rs11986063	rs12953717	rs1321311	rs13254738	rs1447295	rs16892766	rs16901979	rs1862748	rs1957636	rs355527	rs3802842	rs3824999	rs4444235	rs4464148	rs4779584	rs4813802	rs4925386	rs4939827	rs5934683	rs6687758	rs6691170	rs6983267	rs6983626	rs7013278	rs7014346	rs70195668	rs7136702	rs719725	rs7259371	rs7837328	rs961253	rs9929218
Abuli[17]	1,235	OS		**x**			**+**										**x**					**x**					**x**		**x**				**x**									**x**	**+**
Cicek[23]γ	460	OS, DFS													**x**	**x**		**x**															**x**										
Dai[18]γ	285	OS, R	-	**x**	-	**x**	**x**	**x**			**x**	**x**	**x**				**x**		**x**		**+**	**x**		**x**	-				**x**				-	**x**		**x**				**x**	**x**	**+**	**x**
Garcia-Albeniz[24]§	1,509	OS																											-														
Hoskins[19]	583	OS	**x**		**x**		**x**															**x**			**x**	**x**			**x**				**x**		**x**	**x**			**x**				
Passarelli[32]	727	CSS																							**x**				**x**														
Phipps[20]§	2,611	OS, CSS		**x**			**x**		**x**	**x**							**x**					**x**		**x**		**x**		**x**	-		**x**	**x**	**x**					**x**				**x**	**x**
Tenesa[21]	2,838	CSS		**x**			**x**										**x**					**x**		**x**		**x**			**x**				**x**									**x**	**x**
Xing[22]	380	OS, DFS					**x**						**x**									**x**				**x**			**x**								**x**						

Key: + significantly increased survival,—significantly decreased survival, X no survival difference observed

OS—Overall survival, DFS—Disease free survival, CSS—cancer specific survival, R—recurrence

§—Based on overlapping cohort of patients

γ- Based on patients with stage II/III cancers.

**Fig 3:**
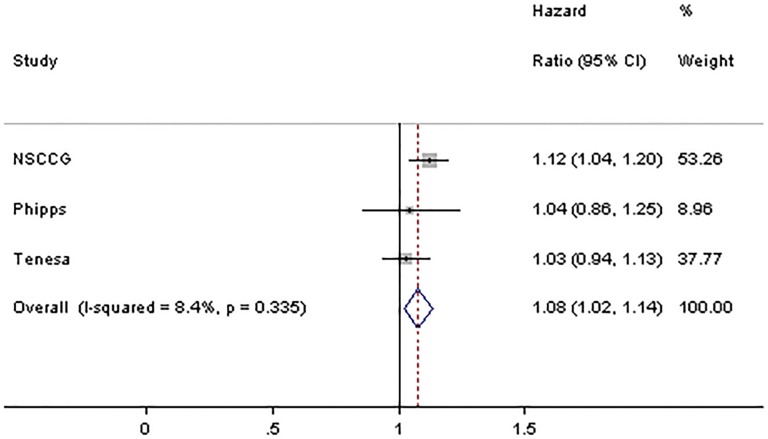
Forest plot showing the results of a meta-analysis combining HR for the SNP rs4444235. Horizontal lines represent 95% confidence intervals. Each box represents the OR point estimate and its area is proportional to the weight of the study. The diamond (and unbroken line) denotes the overall summary estimate, with CIs given by its width. The unbroken vertical line is at the null value (OR = 1.0).

Only two of the previously reported studies have investigated the influence of rs4444235 on prognosis and they found no significant association with survival [20,21]. To further examine the association between rs4444235 genotype and OS a meta-analysis pooling our study with these studies was undertaken (Fig. 3). Collectively, the three studies provided rs4444235 genotypes on a total of 9,686 CRC patients. Using these data, the summary OR was 1.08 (95%CI 1.02–1.14) with the potential of heterogeneity between studies (*P*_*het*_ = 0.34; I^2^ = 8%).

## DISCUSSION

Here we have provided evidence that variation at 14q22.2 defined by rs4444235 influences CRC outcome independent of established metrics. Although our study did not provide evidence for a relationship between other SNPs our analysis only had 50–70% power to demonstrate a relationship between carrier status for a 10% difference in prognosis, at the 5% threshold. Hence it is not possible to conclusively exclude the possibility that variation at the other CRC risk loci may also be linked to outcome.

Major strengths of our study are its size, the fact that it is drawn from a representative sample of the population, and involved the systematic follow-up of patients. Overall survivorship is unlikely to have influenced study findings, even though case selection in NSCCG is biased to Dukes’ stages A and B disease. It therefore seems unlikely that any spurious influences as a consequence of study design will have impacted significantly on our findings. Furthermore, as our analysis was restricted to UK patients with self-reported European ethnicity our study findings are also unlikely to be confounded by population stratification.

We do however acknowledge that a limitation of our study is that we have not addressed potential bias arising from non-uniform treatment. While this is a potential serious confounder in studies of some tumours the management of CRC is relatively uniform within the UK. Support for this assertion is provided by the fact that survival rates observed in our study population were not different to those expected of other unselected patients of a similar stage profile treated in the UK [2]. It is likely that the impact of risk variants will be contingent upon interaction with non-genetic risk factors. Unfortunately, such data were not available within the current study to allow such an analysis.

Mechanistically a functional basis for only the 14q22.2 association has yet to be fully elucidated. It is also noteworthy that the risk allele of rs4444235 appears to be preferentially associated with the development of microsatellite stable CRC[29,30]. This is consistent with the observation that germline mutation in the TGF-β superfamily-signalling pathway genes is associated with microsatellite stable CRC, and hence may impact indirectly on patient outcome. Furthermore, reporter gene studies have demonstrated that the element to which rs4444235 maps acts as an allele-specific transcriptional enhancer. Allele-specific expression studies in CRC cell lines heterozygous for rs4444235 have shown significantly increased expression of bone morphogenetic protein-4 (BMP4) associated with the risk allele providing evidence for a functional basis for the non-coding risk variant [31].

This analysis has provided evidence that variation in 14q22.2 plays a role in defining individual patient prognosis. However compelling this association between 14q22.2 and OS is on the basis of biological plausibility as with all association studies independent validation of study findings is required. While previously published studies have not provided support for the 14q22.2 association such studies are small and hence have had limited power to demonstrate a relationship[17–24,32]. Hence our analysis serves to highlight the statistical problem of searching for genetic associations when the impact of any variant is likely to be at best modest. Even stipulating significance level of 0.05 for an analysis of clinical trial data is unrealistic, because to have 80% power to demonstrate a 5% difference in survival, which is clinically relevant, requires at least 4,800 patient samples to be analysed, even if the frequency of the at risk genotype is 50%. Hence it is therefore not perhaps surprising that previously purported associations cannot be considered robust if FPRP type criteria are imposed.

While germline variants are unlikely to replace staging schemes and conventional markers, they have potential to assist in distinguishing different outcome patterns among patients with the same stage disease where 10% differences are clinically relevant thereby opening up the possibility of a rational, targeted approach to treatment based on a combination of genotype and tumour characteristics of a patient.
